# Novel method of biopsy for a bile duct lesion in the hepatic hilum with a new slim cholangioscope using a tapered-tip sheath

**DOI:** 10.1055/a-2535-8610

**Published:** 2025-03-03

**Authors:** Kojiro Tanoue, Hirotsugu Maruyama, Yoshinori Shimamoto, Tatsuya Kurokawa, Yuki Ishikawa-Kakiya, Akira Higashimori, Yasuhiro Fujiwara

**Affiliations:** 1 Department of Gastroenterology, Osaka Metropolitan University Graduate School of Medicine, Osaka, Japan

Recently, several studies have reported the efficacy of a new slim cholangioscope in endoscopic retrograde cholangiopancreatography (ERCP) 1 2 3 4 . This cholangioscope is notable for its ease of insertion into narrow bile ducts compared with conventional cholangioscopes. However, one limitation is that cholangioscopy-guided biopsy cannot be performed due to the absence of a forceps channel. We present a novel biopsy method for bile duct lesions in the hepatic hilum with a new slim cholangioscope (DRES Slim Scope and CMOS Camera; Japan Lifeline Co. Ltd., Tokyo, Japan) using a tapered-tip sheath (ERCP Guide Sheath; Olympus, Tokyo, Japan).

A 66-year-old woman underwent ERCP for transient jaundice. Based on cholangiography and intraductal ultrasonography findings ( Fig. 1 ), we suspected cholangiocarcinoma in the hepatic hilum and attempted to insert a conventional cholangioscope; however, we could not because the distal bile duct was narrow. Histopathological analysis of the tissues obtained through transpapillary biopsy under fluoroscopy revealed atypical cells. The patient underwent ERCP again. After placement of the guidewire in the deep bile duct, a slim cholangioscope ( Fig. 2 ) was inserted into the bile duct near the mass in the hepatic hilum. The mass was confirmed by cholangioscopy to be a polyp-like lesion. Subsequently, the cholangioscope was removed, and a tapered-tip sheath ( Fig. 3 ) was inserted into the hepatic hilum. Through the sheath, the thin camera of the cholangioscope was successfully advanced together with a small biopsy forceps (SpyBite Max; Boston Scientific, Marlborough, Massachusetts), allowing tissue collection via cholangioscopy-guided biopsy ( Video 1 ).

**Fig. 1:**
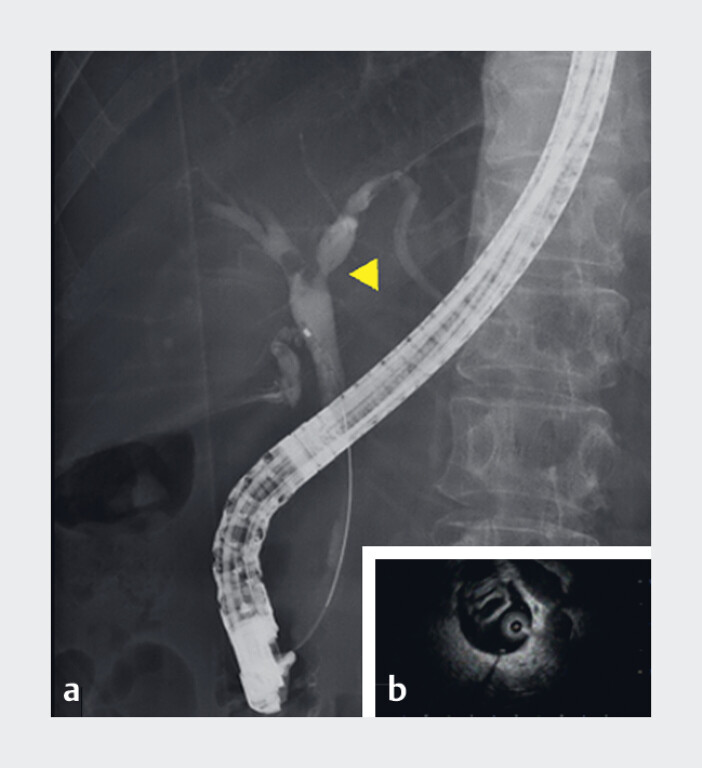
Findings revealing a tumor in the bifurcation of the right and left hepatic ducts. **a** Endoscopic retrograde cholangiography. **b** Intraductal ultrasonography.

**Fig. 2:**
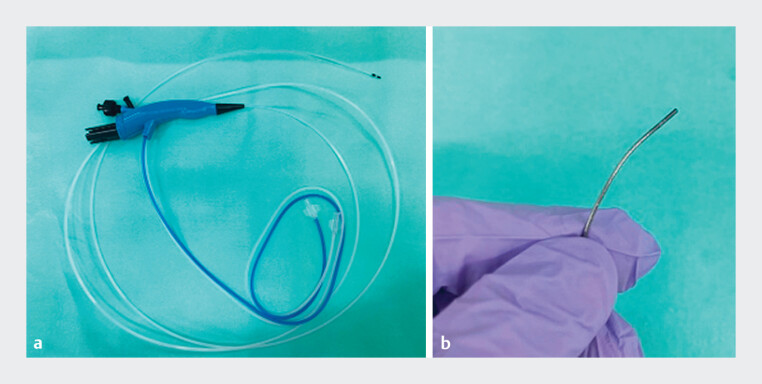
A new slim cholangioscope. **a** A slim scope with a diameter of 2.6 mm and a length of 1,950 mm. **b** A thin camera with a diameter of 1.0 mm and a length of 2,100 mm.

**Fig. 3:**
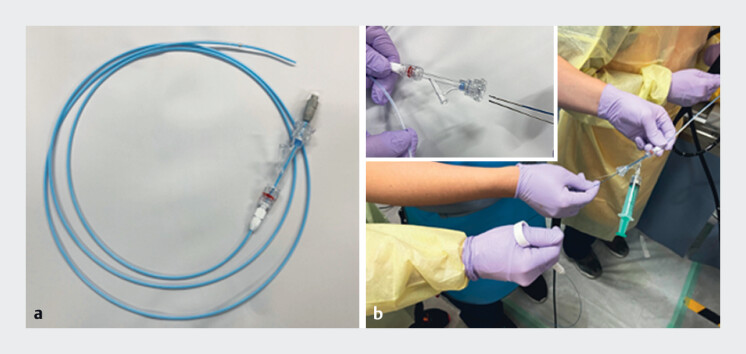
A tapered-tip sheath and the novel method. **a** A device including an inner catheter with a tapered tip and an outer sheath with a diameter of 8.5 Fr (2.8 mm). **b** Photograph showing insertion of the thin camera and a small biopsy forceps through the sheath.

Novel method of biopsy for a bile duct lesion in the hepatic hilum with a new slim cholangioscope using a tapered-tip sheath.Video 1

Histopathological examination revealed inflammatory granulation tissue and no malignancy ( Fig. 4 ). By combining this thin camera with the sheath, a direct visual biopsy can be performed. This method enables biopsy in challenging areas and helps to reduce stress on endoscopists due to the thinness of the cholangioscope. This novel approach could be a breakthrough in further examination of the bile duct.

**Fig. 4:**
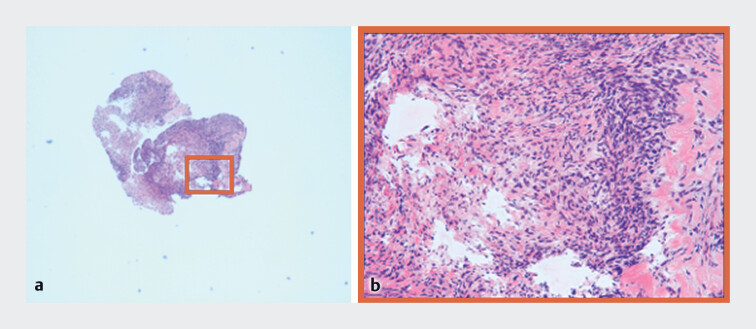
Histopathological result by cholangioscopy-guided biopsy. Images showing hematoxylin and eosin staining. **a** Low power field. **b** High power field.

Endoscopy_UCTN_Code_TTT_1AR_2AD

## Notes

Endoscopy E-Videos https://eref.thieme.de/e-videos
*E-Videos* is an open access online section of the journal *Endoscopy* , reporting on interesting cases and new techniques in gastroenterological endoscopy. All papers include a high-quality video and are published with a Creative Commons CC-BY license. Endoscopy E-Videos qualify for HINARI discounts and waivers and eligibility is automatically checked during the submission process. We grant 100% waivers to articles whose corresponding authors are based in Group A countries and 50% waivers to those who are based in Group B countries as classified by Research4Life (see: https://www.research4life.org/access/eligibility/ ). This section has its own submission website at https://mc.manuscriptcentral.com/e-videos .
